# An integrated One Health initiative for pathogen genomic surveillance in the UK

**DOI:** 10.1099/mgen.0.001539

**Published:** 2025-11-06

**Authors:** Yogesh K. Gupta, Ian Adams, Ronny van Aerle, Justin Avant, David Bass, Frederico M. Batista, Marco Benucci, Tiernan Briggs, Irene Cano, Bhudipa Choudhury, Bridget Crampton, Richard J. Ellis, Graham Freimanis, Edward Haynes, Sarah C. Hill, Eleanor Jones, Lynn Laurenson, Alastair Maclaren, Dan Maskell, Leone Olivieri, Richard Paley, Oliver G. Pybus, Jayna Raghwani, Helen Roberts, Tahmina Ruba, Pankajini Samal, Mirjam Schilling, Sunitha Subramaniam, Neil Taylor, Georgia M. Ward, Lisa Ward

**Affiliations:** 1 Virology Department, Animal and Plant Health Agency, Woodham Lane, Addlestone, UK; 2 Fera Science Ltd, York Biotech Campus, York, UK; 3 Centre for Environment, Fisheries and Aquaculture Science, Weymouth, UK; 4 Forest Research, Alice Holt Lodge, Wrecclesham, Farnham, UK; 5 Department of Surveillance and Laboratory Services, Animal and Plant Health Agency, Addlestone, UK; 6 The Pirbright Institute, Ash Road, Pirbright, Woking, UK; 7 Department of Pathobiology and Population Sciences, Royal Veterinary College, London, UK; 8 Forest Research, Northern Research Station, Roslin, Midlothian, EH25 9SY, UK; 9 Department of Biology, University of Oxford, Oxford, UK; 10 Department for Environment, Food & Rural Affairs, London, UK; 11 Forest Research, Foss House, York, UK

**Keywords:** biosecurity, epidemiology, genomics, One Health, pathogenomics, surveillance

Genomic surveillance has become an increasingly important component of disease management, enabling precise identification of pathogens, real-time tracking of outbreaks and insights into host–pathogen co-evolution. Technological and analytical advances now permit high-throughput sequencing (HTS) to be undertaken across a wide range of contexts, from large-scale sequencing in genomics centres to on-site sequencing in the field. As the cost of sequencing per sample continues to decline and its accessibility improves, the potential for genomic surveillance to mitigate threats posed by endemic and emerging diseases grows [1,2]. A One Health framework that leverages genomic surveillance for animal and plant pathogens can therefore address critical challenges in agriculture, public health and biosecurity, especially in an era characterized by globalization, climate change and increasing antimicrobial resistance (AMR).

Protecting livestock, crops, fisheries, forestry and aquaculture from endemic and emerging infectious diseases is a global challenge. Animal and plant pathogens and pests can cause economic losses, degrade environments, compromise animal welfare, challenge public health and damage a nation’s international reputation. The UK’s approach to biological hazards is underpinned by the four foundational pillars of its biological security strategy: understand, prevent, detect and respond [3]. Among these, the detection of infectious agents plays a pivotal role as it triggers subsequent actions and interventions. Surveillance, therefore, stands as an early warning tool and first line of defence against endemic and emerging pathogens and pests, enabling timely control efforts to safeguard critical sectors of society. Looking to the future, there will be opportunities for new technologies such as genomics and artificial intelligence to strengthen the predictive components of national biosecurity frameworks, by enabling early risk assessments and proactive interventions.

The UK needs world-leading, risk-based, early warning surveillance systems that integrate passive and active infectious disease surveillance to address present and future risks [4]. Surveillance activities involve the systematic measurement, collation, analysis, interpretation and timely dissemination of data related to animal and plant health within defined populations. Surveillance can be categorized into three broad themes: (1) early warning surveillance, which includes horizon-scanning, international disease monitoring and scanning surveillance for animals and plants; (2) risk-based targeted (active) surveillance, focusing on high-priority pathogens and pests; and (3) surveillance to determine disease-free status, ensuring compliance with trade and regulatory standards [5]. Strategic initiatives such as the UK Biological Security Strategy [3], the Vision for Animal and Plant Health Research [6] and the Plant Biosecurity Strategy for Great Britain [7] have underscored the urgency of strengthening surveillance, particularly to mitigate increasing threats posed by pandemics and other emergent risks.

Addressing these challenges is complex due to the distributed structure of the UK’s animal and plant health science landscape [8]. Responsibility is shared across the four devolved UK administrations: the Department for Environment, Food and Rural Affairs (Defra) in England, the Scottish Government’s Agriculture and Rural Economy Directorate, the Welsh Government’s Economy, Environment and Rural Affairs and the Department of Agriculture, Environment and Rural Affairs in Northern Ireland. These administrations work collaboratively to safeguard the UK’s natural environment, support the food and farming industries and foster a thriving rural economy. Within each administration, governance is shared across multiple departments and agencies, creating an intricate framework that requires coordinated efforts to achieve a cohesive and unified approach.

For instance, Defra oversees a network of 33 agencies and public bodies in England, including key partners in the animal and plant health sectors such as the Animal and Plant Health Agency (APHA), the Rural Payments Agency, the Centre for Environment, Fisheries and Aquaculture Science (CEFAS), the Veterinary Medicines Directorate, Forest Research and the Royal Botanic Gardens, Kew. Further critical contributions come from non-departmental organizations such as the Pirbright Institute and Fera Science Ltd and the UK’s higher education sector. This multi-faceted system creates opportunities for innovation but also risks fragmentation and duplication of efforts, including in the adoption and implementation of HTS technologies such as whole-genome sequencing, metabarcoding and other ‘omics’ for pathogen detection. Coordination across all institutions involved is essential to unlock the full potential of genomic surveillance within the UK and to ensure a cohesive national strategy for managing animal and plant health threats.

There are several global initiatives that exemplify the One Health approach to genomic surveillance. These include the African Field Epidemiology Network [9], which strengthens field epidemiology capacity across Africa; PANDEM [10], a programme focused on pandemic preparedness and response; the European Partnership for Animal Health and Welfare [11], which fosters collaboration among European scientists and funders – including UK partners – to improve animal health; and Preventing Zoonotic Disease Emergence [12], an international initiative aimed at enhancing prevention, early detection and resilience against emerging infectious diseases of animal origin. By engaging with and learning from these programmes, stakeholders worldwide can contribute to and benefit from global efforts to develop integrated and cross-sectoral genomic surveillance systems.

The Genomics for Animal and Plant Disease Consortium (GAP-DC) [13] was launched in July 2023 and is supported by Defra and UKRI. GAP-DC has been designed to cut through some of the abovementioned complexity by bringing together key organizations in the understanding of pathogen detection and genomics for terrestrial and aquatic animal health (APHA, Royal Veterinary College, CEFAS, The Pirbright Institute) and plant health (Fera Science, Forest Research). By fostering stronger collaborations between academic institutions and government agencies, GAP-DC enhances the role of the higher education sector in animal and plant disease surveillance, research and response – bringing academic expertise into closer alignment with national biosecurity priorities. GAP-DC aims to improve disease detection and elucidation, track pathogen transmission and evolution and enhance national and global biosecurity frameworks, providing a coordinated approach in tackling animal and plant health challenges.

The scope of the GAP-DC initiative is defined by six interconnected work packages, each addressing a critical aspect of genomic surveillance. The first work package focuses on enhancing frontline pathogen detection at high-risk locations, such as border control posts, by deploying satellite or mobile laboratory facilities and evaluating HTS technologies. This aim is to improve the rapid detection of pathogens affecting animals, plants and aquatic systems; facilitate timely interventions; and improve biosecurity at the national level.

The second work package targets the spillover of pathogens between wild and farmed/cultivated populations. By detecting pathogens at the interface between wild and domestic environments, it provides a crucial opportunity for early intervention. This work package also addresses limitations in existing methods such as PCR-based detection methods and investigates alternative host or environmental reservoirs that may play a role in plant and aquatic disease dynamics.

The third work package seeks to advance the identification and understanding of the disease agents contributing to syndromic or complex diseases, including both notifiable/regulatory agents and those not currently recognized by legislation or identified as disease-causing agents. This includes syndromic diseases such as post-weaning mortality in pigs, red skin disease of salmon and wasting diseases of seagrasses. Notifiable pathogens including foot-and-mouth disease virus, Bluetongue virus, Lyssavirus, *Cyprinid Herpesvirus* 3, *Phytophthora* spp. and *Sirococcus tsugae* are also included in this work package. By providing detailed genetic insights, GAP-DC will enhance diagnostic accuracy and strengthen surveillance capabilities, whilst ensuring regulatory compliance.

The fourth work package develops frameworks for detecting and managing outbreaks of new and re-emerging diseases and increasing our ability to harness genomic data from complex eukaryotic genomes. By leveraging genomic data, this work will identify markers of virulence, resistance and transmission, enabling swift, evidence-based responses. It also emphasizes collaborative engagement among stakeholders during crisis scenarios to optimize the management and control of outbreaks.

The fifth work package explores innovative strategies for mitigating endemic diseases. By integrating genomic data with epidemiological approaches, it will develop sustainable, long-term management solutions to reduce the burden of endemic diseases on agriculture and public health.

The sixth and final work package focuses on enhancing coordination among key stakeholders, as well as existing and potential end users. Stronger inter-agency partnerships are needed to ensure that evidence-based decisions are supported by comprehensive cost-benefit analyses and stakeholder engagement. This work package actively engages with and builds upon the foundations of other UK pathogen genomic surveillance programmes, such as Pathogen Surveillance in Agriculture, Food and Environment (PATH-SAFE) [14] and the UK Microbial Forensic Consortium (UKMFC) [15]. PATH-SAFE focuses specifically on foodborne pathogens and AMR across agri-food systems, whilst UKMFC is dedicated to microbial forensics, supporting national security and law enforcement through advanced microbial analysis. In contrast, GAP-DC takes a cross-sectoral approach across animal, plant and aquatic health domains to address technological and policy challenges shared among multiple diseases and sectors. It promotes collaboration and knowledge exchange between agencies, disciplines and disease systems and explores innovative methodologies such as environmental metagenomics, which are not currently covered by any existing programmes. Through this broader scope, GAP-DC aims to promote unified responses among agencies and improve public communication during health crises.

To share best practice, utilize new learnings and prevent duplication of effort among the work packages, the GAP-DC initiative employs a cross-cutting approach across the work packages employing four key technical domains. First, ‘Sampling and Sequencing’ ensures robust and representative pathogen detection across diverse hosts and environments. Second, ‘Data Processing and Analysis’ applies bioinformatics pipelines and computational tools to extract meaningful insights from genomic data. Third, ‘Quality Assurance - Validation and Accreditation’ upholds data reliability, reproducibility and compliance with accreditation frameworks. Finally, ‘Evidence-to-Policy’ translates scientific findings into policy development, enabling data-driven decision-making for improved disease surveillance, response and prevention (Fig. 1).

**Fig. 1.**
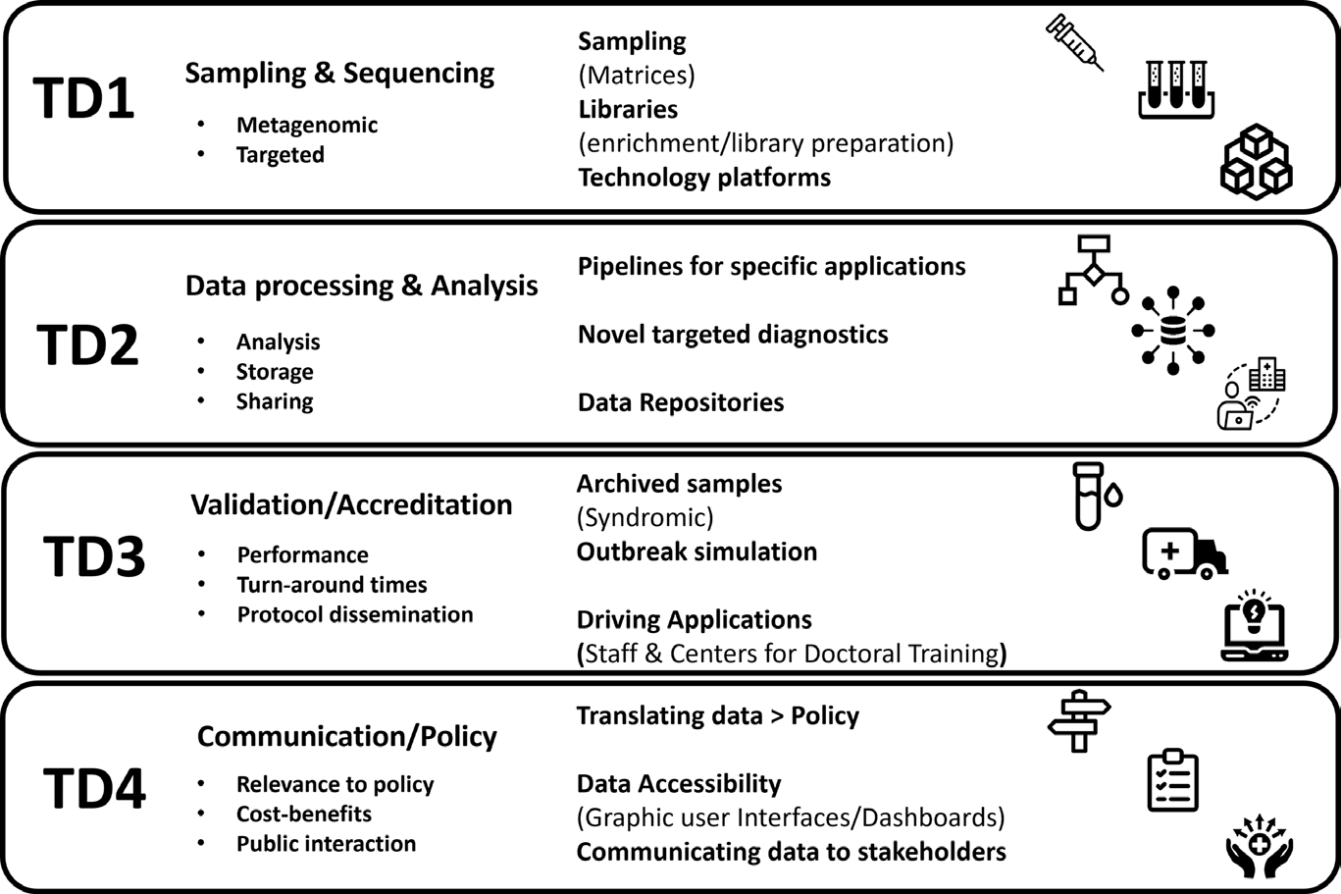
The four key technical domains of the GAP-DC initiative, including (TD1) sampling and sequencing, (TD2) data processing and analysis, (TD3) quality assurance – validation and accreditation – and (TD4) evidence to policy.

GAP-DC focuses on animal, plant and aquatic health whilst also aligning with human health surveillance efforts, thereby contributing to the broader One Health agenda. As mentioned above, GAP-DC engages with programmes such as PATH-SAFE, which have direct relevance to public health. It also aligns with the vision of the National Biosurveillance Network [3], a UK government initiative that adopts a One Health approach to unify surveillance data across human, animal, plant and environmental health to protect against high-consequence biological threats. Collaborations of this kind foster methodological alignment, harmonization of data standards and the development of interoperable bioinformatics tools that span human, animal and plant health domains, ensuring effective coordination with human health agencies such as UK Health Security Agency.

GAP-DC is committed to fostering national and international partnerships to strengthen global pathogen surveillance. By sharing methodologies and data, and developing new approaches, the consortium will establish a robust foundation for collaborative responses to transboundary disease threats. Future priorities include expanding surveillance networks, enhancing bioinformatics capabilities and integrating innovative technologies to address evolving challenges, including building predictive capabilities to better anticipate and mitigate disease impact. Further information, along with a portal for queries, can be found on the project’s website: https://www.gapdc.org.

## Abbreviations

AMR: antimicrobial resistance
APHA: Animal and Plant Health Agency
CEFAS: Centre for Environment, Fisheries and Aquaculture Science
Defra: Department for Environment, Food and Rural Affairs
GAP-DC: Genomics for Animal and Plant Disease Consortium
HTS: high-throughput sequencing
PATH-SAFE: Pathogen Surveillance in Agriculture, Food and Environment
UKMFC: UK Microbial Forensic Consortium
